# The Community as the Unit of Healing: Conceptualizing Social Determinants of Health and Well-Being for Older American Indian Adults

**DOI:** 10.1093/geront/gnac018

**Published:** 2022-01-29

**Authors:** Elise T Jaramillo, Emily Haozous, Cathleen E Willging

**Affiliations:** Pacific Institute for Research and Evaluation, Albuquerque, New Mexico, USA

**Keywords:** American Indians, Health care utilization, Health equity, Health policy, Well-being

## Abstract

**Background and Objectives:**

Multiple racial and social inequities shape health and access to health care for American Indian Elders, who have a lower life expectancy than all other aging populations in the United States. This qualitative study examines how upstream social determinants of health influence Elders’ ability to access and use health care.

**Research Design and Methods:**

Between June 2016 and March 2017, we conducted individual, semistructured interviews with 96 American Indian Elders, aged 55 and older, and 47 professionals involved in planning or delivering care to Elders in 2 states in the U.S. Southwest. Transcripts were analyzed iteratively using grounded theory approaches, including open and focused coding. A group of American Indian Elders and allies called the Seasons of Care Community Action Board guided interpretation and prioritization of findings.

**Results:**

Participants described multiple barriers that hindered Elders’ ability to access health care services and providers, which were largely tied to funding shortages and bureaucratic complexities associated with health care and insurance systems. Where available, community resources bridged service gaps and helped Elders navigate systems.

**Discussion and Implications:**

Longstanding structural inequities for American Indians manifest in barriers to health equity, many of which are situated at the community level. These are compounded by additional disparities affecting older adults, rural residents, and marginalized citizens in general. Findings underscore the importance of health and policy initiatives for American Indian Elders that emphasize the community as the focus of intervention.

## Background and Objectives

Throughout their life courses, older American Indian adults experience the effects of multiple racial and social inequities (Grandbois & Sanders, 2009). These include harmful physical environments, poor working conditions, and inadequate access to education, health care, and social services, as well as the historical legacies of colonization and contemporary forms of racism, discrimination, and social exclusion. More than a decade of literature on the social determinants of health shows that these inequities are directly implicated in longstanding disparities in health and access to health care (World Health Organization, 2008). This study draws on qualitative interviews with American Indian Elders (aged 55 and older) and professionals who work with them in two Southwestern states to examine the question: How do social determinants shape disparities in access to health care for this population? Of note, our use of the word “Elder” is guided by our advisory group of Elders and allies (described below) who prefer this term to respectfully describe older adults in their communities.

### Determinants of Health for American Indian Elders

Social determinants of health frameworks draw attention to the upstream social and economic conditions that shape health and health disparities (Sadana et al., 2016). However, Indigenous scholars have suggested that the concept of “social determinants,” which may encompass a broad array of environmental, economic, and political conditions, may be too general to account for the complex, persistent, and specific effects of colonization, forced acculturation, and racial discrimination in the lives of Indigenous peoples (de Leeuw et al., 2015). These structures comprise root determinants of disruptions of Indigenous health and social structures that resulted from colonization, medical pathologization, social exclusion, and discrimination (Smith, 2012; U.S. Commission on Civil Rights, 2018).

In the United States, these root determinants have shaped inequitable access to adequate health care, which is a crucial factor contributing to health disparities among American Indian Elders (Kim et al., 2012). Inequities in health care access for this population derive from colonial relationships between the U.S. government and Indigenous nations (Skinner, 2016; U.S. Commission on Civil Rights, 2018). The legal right to health care for members of federally recognized American Indian tribes was established in treaties and the U.S. Constitution, with federal appropriations dedicated to American Indian health starting in 1911. These appropriations were formalized by the 1921 Snyder Act and the 1955 Transfer Act, which established the Indian Health Service (IHS; Skinner, 2016). The IHS encompasses a large network of hospitals, clinics, and programs serving 2.56 million American Indians and Alaska Natives (IHS, 2019). These include facilities run entirely by the IHS, programs where tribes have fully or partially assumed control over IHS funds under the IHS Tribal Self-Governance Program, and urban health clinics. Members of federally recognized tribes have access to primary care services at IHS facilities at no cost and without health insurance. Referrals for care not available at the IHS, commonly including specialty care and many diagnostic tests, are made through the Purchased/Referred Care (PRC) program. If American Indians who qualify for the IHS need emergency care unavailable at an IHS or tribal facility, they must report having received this care to the PRC office within 72 h to have it covered.

Yet, federal funding for the IHS is discretionary and chronically inadequate. For example, in the fiscal year 2019, per capita expenditure for the IHS user population was $4,078, compared to $9,726 for the general U.S. population in 2017 (IHS, 2019). In this perpetually underfunded state, IHS facilities are often outdated and short-staffed. At the same time, PRC payments must be tiered and prioritized depending on the amount of funding available (U.S. Commission on Civil Rights, 2018). In addition, although the majority of American Indians live in urban areas, less than 1% of the IHS budget is devoted to urban clinics (National Indian Health Board, 2020). The IHS also does not have a budget line item to fund services that many older adults need, such as in-home health care, assisted living, and other long-term care (Bylander, 2018).

To compensate for these severe funding gaps, IHS and tribal programs depend on reimbursements from Medicare, Medicaid, and private health insurance for their services to IHS patients with health insurance (Bylander, 2017). The IHS is the “payor of last resort,” meaning that it can bill patients’ insurance before using IHS dollars for their care. The 2010 Patient Protection and Affordable Care Act (ACA) included additional provisions to make insurance enrollment easier for American Indians and Alaska Natives, including unlimited enrollment windows and additional cost-sharing arrangements (Skinner, 2016). In states that expanded Medicaid eligibility under the ACA to adults at or below 130% of the Federal Poverty Level, many older American Indians who were not eligible for Medicare (e.g., most of those younger than age 65) were newly able to enroll in Medicaid (Artiga et al., 2017). Many IHS and tribally run programs thus strongly encourage patients to enroll in health insurance. Health insurance also allows patients to seek health care outside the IHS system. Still, many American Indian Elders find the use of health insurance prohibitively burdensome and confusing. They may also have trouble accessing other forms of health care because of geographic distance or limited provider networks, or they simply prefer to use IHS facilities (Jaramillo & Willging, 2021; Sommerfeld et al., 2021).

Our previous publications illuminate various factors impinging on Elders’ senses of wellness and health care experiences, including policy- and system-level influences (Willging et al., 2021), and their pragmatic and affective consequences (Jaramillo & Willging, 2021). The present analysis highlights American Indian Elders’ ability to access health care as a lens to examine how structural determinants create barriers to health equity for this population. In addition, this work responds to calls to reveal how identity (i.e., age, race) affects health and pinpoints the community as a crucial but underexamined point of intervention (Sadana et al., 2016).

## Research Design and Methods

Qualitative research methods are useful for capturing rich, descriptive information about perceptions, beliefs, and experiences (Patton, 2015). These qualitative data were collected as part of a multiyear, mixed-method study exploring experiences with health care and health insurance among American Indian Elders (aged 55 and older) in two states in the Southwestern United States (Willging et al., 2018). The concept for the study was developed collaboratively by non-Indigenous and Indigenous researchers at the request of a group of American Indian Elders and allies who were concerned about the health of Elders in their communities. These individuals became the Seasons of Care Community Action Board (CAB). They have participated in each aspect of the study, including reviewing and testing data collection instruments, assisting with recruitment procedures, and providing feedback on the interpretation and prioritization of findings. Data were collected with the assistance of eight Elder consultants from the communities participating in the study. These consultants received training in data collection and human participants’ protection and then accompanied researchers into the field, helped conduct and debrief interviews, and provided cultural and linguistic translations when necessary. We obtained formal approval from each tribal community in which research was conducted. In the interest of reciprocity, research participants were compensated for their time and knowledge sharing, and many gave feedback on research findings. Given the history of exploitative research practices involving Indigenous people, these community-engaged approaches center participants’ voices and knowledge in the research process, which is in keeping with Indigenous methodologies (Brave Heart et al., 2016; Smith, 2012).

### Participants

To recruit Elder participants, we used a purposive sampling strategy intended to represent the range of views within a group to determine similarities and differences in knowledge, beliefs, and experiences (Patton, 2015). Our sample included 96 American Indian Elders and 47 professionals. To recruit Elder participants, the researchers (including the first and third authors) made regular visits to American Indian senior centers, community health fairs, and meetings of senior-serving organizations to present the study and invite attendees to participate. Eligible participants were aged 55 and older and self-identified as American Indian. Although age 65 is the most common threshold to define older adults, age 55 more appropriately reflects the cumulative effects of historical trauma on reduced life expectancy among American Indians (Palacios & Portillo, 2009). Table 1 outlines the characteristics of Elder participants.

**Table 1.** Characteristics of American Indian Elder Participants (*N* = 96)

Variable	*M*	Range	%	*N*
Age, years	67	55–89		
Gender				
Women			70.8	68
Race/ethnicity				
American Indian			100	96
Hispanic			17.7	17
Spoke an Indigenous language as first language			47.9	46
Education				
High school/general equivalency diploma or less			27.1	26
Some college or vocational training			33.3	32
Associate’s or vocational training degree			22.9	22
Bachelor’s degree			9.4	9
Master’s degree			5.2	5
Doctoral degree			2.1	2

We recruited the professional participants using reputational case selection (LeCompte & Schensul, 2010). The members of the CAB and a state chapter of the National Indian Council on Aging suggested professionals who were involved in planning, administering, and/or delivering health care to Elders. We reached out to candidates via email. Ten tribal leaders (e.g., elected officials), 16 administrators (e.g., IHS personnel, government system administrators), nine health care providers (e.g., doctors, nurses), and 12 outreach workers (e.g., community health representatives [CHRs], benefits coordinators, insurance company liaisons) agreed to participate. Table 2 outlines the characteristics of professional participants.

**Table 2.** Characteristics of Professional Participants (*N* = 47)

Variable	*M*	Range	%	*N*
Age, years	51	28–74		
Gender				
Women			59.6	28
Race/ethnicity				
American Indian			63.8	30
Hispanic			27.7	13
Education				
High school/general equivalency diploma or less			6.4	3
Some college or associate’s degree			27.4	13
Bachelor’s degree			25.5	12
Master’s degree			19.1	9
Doctoral degree			21.3	10
Years worked at the current organization	9.6	0.4–33		
Years worked in the current occupational field	19.5	1.5–42		
Years worked in or with American Indian communities	20.8	1.3–57		

All participants signed a written informed consent document according to which identifying features were withheld from the data reported below. The research was approved by the Southwest Tribal Institutional Review Board. Participants were offered $25 for their involvement.

### Data Collection

The Elders were interviewed by experienced researchers paired with an American Indian Elder consultant. Interviews were administered in English, although the Elder consultants were available to perform the interview in the Indigenous languages of participants preferring this option. Interviews included a quantitative survey to gather information about demographics, health status, health insurance, and health care access and utilization. We then followed a semistructured interview guide with a series of open-ended questions about Elders’ experiences and perceptions of wellness, health care, and health insurance, including factors influencing their decisions about help-seeking and care, satisfaction with care, and knowledge of and experience with the ACA, Medicare, Medicaid, and other insurance programs. The interviews typically lasted between 60 and 90 min and were conducted in a setting deemed private and convenient by the Elder, including homes and private rooms located in senior centers. After each interview, researchers and elder consultants engaged in a debriefing conversation to identify important questions and themes, troubleshoot problems, and discuss any cultural or historical information that researchers may not have known.

Interviews with professionals consisted of open-ended questions centered on participant work roles and responsibilities, background and experiences working with Elders, knowledge of factors affecting health care and health insurance for Elders, perceptions of how national and state reforms affect Elders, and recommendations for enhancing access to services and overcoming insurance barriers. Most interviews occurred in person, usually at the participant’s place of work; eight were conducted over the phone. The interviews lasted 45–60 min. All interviews were digitally recorded and professionally transcribed for analysis.

### Data Analysis

Data were analyzed using grounded theory methodology, an iterative approach to analysis and comparison that allows key findings to emerge from textual data (Charmaz, 2014). Employing a constant comparative process, four team members reviewed the transcripts to develop and agree upon a coding scheme that reflected the different issues concerning health care and insurance for American Indian Elders. We assigned codes to segments of text ranging from a phrase to several paragraphs based on the coding scheme. Next, we created additional codes based on sensitizing concepts related to health care access (e.g., appointments, waiting, transportation). These concepts provided “a general sense of reference” and supplied descriptive data based on the words of Elders and professionals, enabling us to examine their salience and meaning (Patton, 2015, p. 545). We then engaged in open coding to identify and define new codes that we had not previously considered (e.g., provider relationships, language), followed by focused coding to determine which themes/issues recurred or represented unusual concerns to participants (Corbin & Strauss, 2008). By constantly comparing and contrasting codes with one another, we grouped codes with similar content or meaning into broader themes linked to segments of transcript text (Corbin & Strauss, 2008). We created a detailed outline describing and linking codes to each theme/issue and reviewed this work collectively. Discrepancies in coding and analysis were identified during this process and resolved during team meetings. Summary reports of key themes/issues were shared and discussed with the CAB and at meetings at senior centers and wellness centers in the communities of study. Our results were thus generated through an iterative process of drawing out themes and sharing them with participants and the CAB for guidance.

## Results

The Elders we interviewed came from more than a dozen tribal backgrounds and lived in both reservation and nonreservation settings across a geographic region of nearly 100,000 square miles, including both rural and urban areas. Still, the majority (88.5%) reported that they used the IHS or another tribally operated program for their care some of the time, and two thirds (66.7%) indicated that the IHS was their usual source of care. In addition, nearly all (97.9%) reported that they were enrolled in health insurance, most commonly Medicare and/or Medicaid. However, despite having health insurance, Elders and professionals described a variety of issues that often made it difficult for Elders to access care. As described below, these issues fell into two broad themes: (a) access to health care services and (b) access to health care providers. A third theme, access to community programs, captures some of the solutions tribes employed to bridge common gaps in services for Elders.

### Access to Health Care Services

Most of the Elders we interviewed had received care from the IHS across their life span. Some described a time in their memory when their local IHS facility offered most of the care they needed, including maternity and inpatient stays. They lamented that these services were no longer available within their community. One 67-year-old man recalled, “[At] the IHS, we had a good hospital, and then … everything fell apart …. We had surgeons, we had the OB [obstetrics], we had rooms for patients, everything.” A woman in her 70s commented, “You used to be able to go to the hospital to get care … and if you needed to be admitted then there was a ward that you could get admitted to, but now there’s only a few beds.” Others listed several types of care that were vital to Elders that the IHS could not provide, such as home health care and caregiver support. Both Elders and professionals also pointed to the lack of assisted living and nursing home options in tribal communities as a major concern for Elders whose families could not support them at home. A second woman in her 70s explained, “There’s tons of things that we want for our Elders, such as nursing homes. We should have our own for Elders here. What’s going to happen when I can’t walk no more? I don’t want to be put in a nursing home [somewhere else].” Those who had the financial resources for an assisted living or nursing home facility were thus usually faced with the prospect of leaving their home communities.

Elders’ access to available services at IHS and tribal facilities was often limited by truncated service hours and long wait times. Elders and professionals observed that there were very few options for Elders who needed urgent care, as no local clinics were open after hours or on weekends. Most health care facilities did not offer same-day or walk-in appointments. Echoing the comments of others, one 70-year-old woman stated, “The times I’ve had to go to urgent care, I would go there [IHS hospital], but they don’t see walk-ins and they can’t do same-day appointments.” Long waits—both to get an appointment and to see a provider once they arrived for an appointment—were the norm for over half of the Elders we interviewed. Elders were thus sometimes obliged to deliberate over whether their need for care outweighed the inconvenience of seeking it. A 61-year-old woman described these deliberations: “I would like there to be more walk-in appointments instead of having to go to the ER [emergency room], but it would never go through. You can go to the doctor in [nearby town], but then you have to start over, so I try to stay away and just heal on my own.”

As this woman’s comment attests, Elders did have access to specialty services and urgent care elsewhere (in theory), either through their health insurance or through PRC. Yet, several obstacles made it difficult for Elders to take advantage of those options. About a third of Elders lived in remote reservations, at least an hour from other health care facilities. For those who could not rely on a family member with a car having the time to drive them to and from appointments, transportation options were sparse, unreliable, or expensive. Although Elders could have utilized transportation assistance or reimbursement through their health insurance, they were often unaware of, or hesitant to use these options, for reasons described below. A health advocate commented, “Many of our Elders have to drive distances to get to the doctor or even to a simple little clinic, and … sometimes it gets a little complicated in terms of if there’s a specialist that they need to travel to, and often they’re [the specialist] in an urban hub, so then that’s added travel time for them and transportation is a huge issue.”

Moreover, some Elders were wary of the care they might receive away from their home community. Interpretation was not always available for the oldest Elders, who were less likely to be fluent in English. Those individuals who could not depend on a family member to provide translation were often at a significant disadvantage. For example, a health care administrator recalled witnessing an older hospitalized woman struggling to make herself understood to her doctors using a translation machine:

She was scared. She was very quiet. She would kind of whisper and so the machine wouldn’t pick it up, so they would be pushing the machine closer and closer to her to be able to translate, which was a nightmare …. I know they felt like these machines really worked well, but I have to tell you from the other end, it was terrifying for this woman.

Finally, Elders were sometimes deterred from seeking care outside of their local IHS or tribal clinic by difficulties associated with getting the costs of their care covered by either PRC funds or health insurance. Elders were familiar with the chronic funding shortages affecting the PRC program. When asked whether they would be confident that PRC would cover the costs of their care if they needed it, Elders’ answers included “I’m not,” “Probably they wouldn’t,” and “It’s hard to say.” In addition, Elders were concerned about running afoul of stringent requirements for reporting care received outside of the IHS to their PRC office and thus ending up with a bill they could not pay. Similarly, although most Elders we interviewed were enrolled in a health plan, some reported that they were hesitant to rely on it because they were unsure what it would cover. In one exemplary comment, a 64-year-old woman explained, “Having used IHS all my life, I’ve never had to deal with health insurance. I kind of don’t want to use it because I might end up with a bill because I don’t know what it’s all about.” Others were deterred from using their health insurance to find care by the need to engage with technology that was unfamiliar or off-putting. An outreach worker who assisted Elders noted that, “It’s becoming increasingly harder [for Elders] to get their questions answered because [insurance plans] are kind of moving everything through technology.”

### Access to Health Care Providers

Despite these struggles accessing health care services, most of the Elders we interviewed spoke highly of their health care providers. However, when Elders did not have a reliable and trusting relationship with a health care provider, they struggled to get the care that they needed. The most common problem that Elders faced in maintaining a relationship with a health care provider was the widespread issue of provider turnover within the IHS. For example, one 59-year-old man commented, “There always seems to be a transition or doctors moving in and out of the program.” Like the gaps in services that Elders experienced, provider turnover was also largely due to funding shortages, which led many IHS and tribal programs to rely on a supply of providers serving short-term stints in their facilities as part of a loan repayment program. Other Elders suspected that providers moved on so quickly because they were unhappy with the economic and educational opportunities in often-remote tribal communities. Whatever the reason for the turnover, some Elders were keenly aware that their providers were not around for the long term. A 65-year-old woman described how she asserted herself to learn how long a provider would stay around from the outset: “We have no choice; they’re going to be changing. That’s why I always ask them, ‘How long are you going to be here? Tell me the truth now.’”

Although they lamented the lack of a long-term, trustworthy relationship with a health care provider, Elders generally reported that they were satisfied with the providers they had. However, some expressed concerns that marred the quality of their interactions with providers. Elders sometimes struggled to communicate with providers, often because they relied too heavily on medical jargon or lacked the cultural understanding to converse clearly and respectfully with Elders. One woman in her 60s recalled being insulted by a physician’s assistant who was measuring her blood sugar level: “My blood sugar was high, and he said, ‘Well I don’t think that’s high because the two hundreds is normal for Indians.’ I just thought that was a horrible statement to make.” They also complained about rushed appointments and providers who seemed more interested in their computers than their patients. Professionals noted that providers sometimes lacked the knowledge and experience about health care and health insurance options available to Elders and American Indians specifically. Other Elders worried about whether providers who were “outsiders” to their community could truly understand their concerns. Finally, professionals again pointed to chronic funding shortages as they expressed concern about the reliance of many programs on short-term contracts with relatively inexperienced providers and unclear credentialing requirements. They worried that such measures compromised the quality of care available to Elders and their families.

### Access to Community Programs

Although most Elders experienced barriers to health care, Elders and professionals also described specific supports that tribal communities provided to help bridge gaps in providers and services. Many Elders relied on benefits coordinators, CHRs, public health nurses, and other tribal outreach workers to help them coordinate their care and move effectively through health care and health insurance systems. Nurses and CHRs interacted with Elders daily as they made home visits, stopped by local senior centers to measure blood pressure and blood sugar levels, and drove Elders to appointments both near and far. For their part, benefits coordinators helped Elders negotiate the often-complex steps associated with navigating the state health insurance exchange or qualifying for Medicaid benefits. Elders often turned to them when they received an unexpected medical bill or needed a referral that they did not know how to get. Commonly employed within their own or a nearby community, these individuals were intimately familiar with the health issues, systemic barriers, and social and cultural norms of the Elders they served. For example, a CHR working for a tribe stated, “I’m traditional. I do participate in our traditional dances [and] my community. I speak the language … so I can speak to my Elders, and I do have the very most respect for my Elders here.” An outreach worker employed by a managed care organization explained, “I was born in an IHS facility. A lot of my healthcare was taken care … at IHS, whether in [small town] or here in [city], it was IHS.” They also served an important role in translating Elders’ values and expectations to their health care providers. A different CHR working for a tribe described an experience advising a doctor on how to speak with an Elder about an amputation in a way that acknowledged the cultural and spiritual significance of the body: “‘Hey doc, you might want to be a little bit sensitive about how you tell them you’re going to remove their toe and you’re making sure you’re telling them you’re not just throwing it [away] or ask them what they want to do with it,’ … like making sure they explain the best care for them but to be really sensitive with the culture.”

Most tribal communities also offered programs, such as tribal ride services, that Elders could use to travel to their distant appointments. Others organized education and outreach efforts to get Elders enrolled in health insurance or raise awareness about health issues, like diabetes. After federal and state funding for benefits coordination under the ACA was cut, some tribes used their financial resources to “[pick] up the tab” to continue those efforts within their community. Others used their funding to pay for additional services, such as care coordination, or cover the premiums of tribal members’ health insurance plans. However, tribes varied widely in their ability to provide such support, often depending on whether they had a significant source of revenue (e.g., gaming) to devote to such services.

## Discussion and Implications

American Indians who are members of federally recognized tribes have a treaty-guaranteed right to health care (Skinner, 2016). The majority of the American Indian Elders in this study were accustomed to exercising this right by relying on IHS or tribal health care providers for at least some of their health care needs. Yet, our findings highlight the numerous barriers that compromised their ability to access the care they needed, from service and provider shortages at their local clinics to the bureaucratic complexities that hindered them from seeking care elsewhere. A social determinant of health approach draws attention to these institutional and systemic limitations—and their foundations in longstanding inequities for American Indian people—as part of the complex web of upstream factors that shape health for aging adults (Sadana et al., 2016). Scholars of American Indian health care highlight the colonial origins of these inequities, which are rooted in the fundamental failure of the U.S. government to honor its trust responsibility to American Indian people by adequately funding IHS and tribal programs (U.S. Commission on Civil Rights, 2018). Our findings detailed the consequences of this unremitted funding shortage for Elders who rely on the IHS: lack of health care services, truncated service hours, long wait times, rushed appointments, and reliance on student loan repayment programs to supply providers who were quick to move elsewhere.

These findings are in keeping with other studies of health care access among American Indians using the IHS (IHS, 2016). However, our data also reveal how additional structural determinants of health compounded Elders’ challenges in accessing health care services and providers (Table 3). Elders experienced common disparities affecting older adults, including the ubiquity of digital technology in managing health care and insurance that Elders struggled to navigate (Intahchomphoo, 2018). Moreover, providers’ lack of linguistic and cultural fluency was doubly burdensome on Elders, who were less likely to be comfortable conversing in English. Similarly, many of the service gaps in tribal communities involved services that Elders most needed, such as assisted living facilities, in-home care, and specialty care for the complex health conditions that older adults are more likely to have (Jacobs et al., 2019). Additionally, service gaps affecting all American Indians, such as long wait times and lack of urgent care, are especially burdensome for Elders, who are likely to need more frequent and complex medical services as they age (Goins et al., 2007).

**Table 3.** Structural Determinants of Health Affecting American Indian Elders’ Access to Health Care

Structural determinants	Resulting factors affecting American Indian elders
Colonial relationship between U.S. government and tribes Self-determination/barriers to self-determination for tribes Discrimination against American Indian people	Underfunding of the Indian Health Service • Provider shortages, turnover, reliance on short-term contracts • Distance to care • Lack of services • Purchased/referred care shortages • Truncated hours • Outdated facilities Culturally incongruent care • Lack of language competence/interpretation • Lack of cultural knowledge Community supports (community health representatives, transportation programs) Cultural knowledge
Underrepresentation of, and discrimination against, older adults	Ubiquity of digital technology Culturally incongruent care • Lack of language competence/interpretation • Lack of cultural knowledge Lack of aging-related services (assisted living, specialty care) Lack of outreach for older adults
Underrepresentation of rural areas	Provider shortages, turnover, reliance on short-term contracts Distance to care
Ideological efforts to erode support for public programs	Bureaucratic complexity associated with navigating health care and insurance systems

Second, some reservation-dwelling Elders’ challenges accessing health care may have been intensified by structural barriers associated with rurality (Winterton et al., 2016). Although provider turnover is a common issue among IHS facilities regardless of location, participants suggested that shortages in health care providers might be tied to dwindling economic and educational opportunities in their rural communities. In fact, provider scarcity and turnover are widespread problems in rural areas (Winterton et al., 2016). When care was not available at local IHS or tribal facilities, geographic and social isolation combined with a lack of transportation options aggravated the difficulty of accessing alternative health care options for rural-dwelling Elders.

Third, many Elders were deterred from accessing care beyond the services available at the IHS by the substantial bureaucratic complexity associated with using their health insurance plans or navigating PRC referral processes. Such bureaucratic burdens, combined with low health literacy (i.e., the ability to locate, understand, and use information about health and health care) stemming from inequities in educational opportunity (Brega et al., 2012), commonly exclude marginalized groups from accessing programs and resources that they need. Although these complexities are often considered unfortunate but necessary, they originate in federal and state rules and regulations and are often tied to political efforts to erode public programs (Herd & Moynihan, 2018). The experiences of Elders in this study underscore how multiple structural barriers—including those affecting all older adults, many rural residents, and other marginalized populations who rely on public programs—overlap to exacerbate the severe inequities that already shape American Indians’ use of health care.

Finally, this research highlights how these structural determinants of health are manifested at the community level, meaning that they specifically affected the community resources available to Elders (e.g., transportation options, culturally congruent care, services, and providers at local IHS or tribal facilities). Moreover, both Elders and professionals emphasized the importance of community supports in bridging the gaps and helping Elders navigate the complexities of health care and insurance systems to get the care they needed. Professionals rooted in the community, like CHRs and benefits coordinators, understood Elders’ needs and values and were able to cultivate this understanding among other professionals. When tribes could afford to provide community programs, like ride services and outreach efforts, these provided resources that Elders generally liked and trusted. In summary, the community was where barriers to care were most keenly felt and the source of many valuable and effective supports. As we have described elsewhere, this resonates with the significance of the community in Elders’ concepts of wellness and concerns about the future (Jaramillo et al., 2019). Indeed, both Elders and professionals often articulated a sense of collective but community-specific experience and perception, such as repeated references to “our Elders.”

Per our prior work, a multilevel approach is necessary to increase health equity for American Indian Elders (Jaramillo & Willging, 2021; Sommerfeld et al., 2021; Willging et al., 2021), including culturally relevant navigation programs to help them move through health and insurance systems, system- and organization-level changes to the way appointments are scheduled and managed, and most importantly, increased and reliable federal funding to supply a full complement of high-quality services. Similarly, the predominance of scholarship on health disparities affecting this population points to the need for direct and reliable funding of health care services for American Indians (Skinner, 2016; U.S. Commission on Civil Rights, 2018). However, data from this study also point to the vital importance of the community as a locus of intervention. While policy- and system-level solutions remain difficult to enact, community-based and -driven programs to support aging tribal members, such as transportation, meal preparation and delivery, and housing support, can go a long way to improve Elders’ health and well-being (Goins et al., 2019).

Federal and state policymakers should thus prioritize working in consultation with tribes, honoring the sovereign rights of tribal communities and supporting the local knowledge of tribal stakeholders as they design tailored programs for their Elders. Policymakers can invest in workforce development and retention efforts in tribal communities and rural areas so that tribal facilities do not have to rely so heavily on short-term student loan repayment programs to supply health care providers. They can also provide additional support for often-overlooked local paraprofessionals, such as CHRs rooted in their communities and able to provide much of the safety net on which Elders rely. Tribal leaders can draw on community-wide health interventions that have been effective in increasing health equity in physical health and social well-being, such as the creation of “health zones” where community residents work to cultivate leadership and align local resources around the root causes of health inequities (Pies et al., 2016). They can also center Elders’ perspectives by engaging them in deliberative dialogs to help plan shared spaces and resources (Canham et al., 2018). Future research can inform efforts among tribal leaders to design and implement adequate and sustainable funding models for such programs. There is also a need to strengthen the connections between health care providers and tribal communities to encourage longevity and cultural knowledge. For example, Elders and health professionals can together take part in bidirectional knowledge sharing learning circles (Wallerstein & Duran, 2010) concerning aging issues and local contexts, including histories (e.g., colonization), vulnerabilities (e.g., lack of transportation), and resources (e.g., local support groups; Goodkind et al., 2014). As a complement to policy advocacy pursuing the full funding of the IHS, such approaches are needed to both reduce the health disparities affecting American Indian Elders and to cultivate supportive environments for them as they age (Sadana et al., 2016).

### Limitations

Due to the substantial diversity of American Indian populations, our sample had several characteristics that may not be generalizable to other groups. Notably, the percentage of Elders in this study who used IHS or tribal health services was much higher than the national average. Our sample also included many reservation-dwelling Elders and Elders who did not speak English as their first language. Their experiences and perspectives may not speak to the challenges facing the majority of American Indians who live in urban areas. We likely oversampled Elders who were more likely to access health care and willing to discuss health with researchers, most of whom were women. Although an American Indian Elder consultant was available to provide linguistic translation as needed, candidates who were not completely comfortable with English may have declined to participate in this research. The involvement of our CAB members—each with decades of experience with Elder health issues—in reviewing findings and providing context likely mitigated some issues with underrepresentation.

## Conclusion

There is an urgent public health need to address the social determinants of severe and persistent inequities in health and health care access affecting American Indian Elders. Although approaches targeting both federal policy and individual-level health behavior and knowledge are crucial, our research emphasizes the community as the locus of multiple barriers rooted in structural determinants of health, as well as of important mechanisms of well-being that can be strengthened to reduce the impact of severe inequity and create a supportive health care environment for older American Indian adults to age healthfully. Consequently, efforts to improve health equity for American Indian Elders must move beyond individuals and health systems to center the community as the unit of healing.
